# Design and Control of Upper Limb Rehabilitation Training Robot Based on a Magnetorheological Joint Damper

**DOI:** 10.3390/mi15030301

**Published:** 2024-02-22

**Authors:** Jintao Zhu, Hongsheng Hu, Wei Zhao, Jiabin Yang, Qing Ouyang

**Affiliations:** 1 College of Mechanical Engineering, Zhejiang University of Technology, Hangzhou 310023, China; jintao_zhu9085@163.com; 2 College of Information Science and Engineering, Jiaxing University, Jiaxing 314001, China; zhaooweii@sina.com (W.Z.); yangjiabin@zjxu.edu.cn (J.Y.); 3 School of Mechanical Engineering, Nanjing University of Science and Technology, Nanjing 210044, China; 4 Taizhou Jiuju Technology Co., Ltd., Taizhou 318000, China

**Keywords:** upper limb rehabilitation robot, magnetorheological joint damper, rehabilitation training, trajectory tracking control, safety testing

## Abstract

In recent years, rehabilitation robots have been developed and used in rehabilitation training for patients with hemiplegia. In this paper, a rehabilitation training robot with variable damping is designed to train patients with hemiplegia to recover upper limb function. Firstly, a magnetorheological joint damper (MR joint damper) is designed for the rehabilitation training robot, and its structural design and dynamic model are tested theoretically and experimentally. Secondly, the rehabilitation robot is simplified into a spring-damping system, and the rehabilitation training controller for human movement is designed. The rehabilitation robot dynamically adjusts the excitation current according to the feedback speed and human–machine interaction torque, so that the rehabilitation robot always outputs a stable torque. The magnetorheological joint damper acts as a clutch to transmit torque safely and stably to the robot joint. Finally, the upper limb rehabilitation device is tested. The expected torque is set to 20 N, and the average value of the output expected torque during operation is 20.02 N, and the standard deviation is 0.635 N. The output torque has good stability. A fast (0.5 s) response can be achieved in response to a sudden motor speed change, and the average expected output torque is 20.38 N and the standard deviation is 0.645 N, which can still maintain the stability of the output torque.

## 1. Introduction

Various dysfunctions often occur after stroke, and hemiparesis is the most common type of motor dysfunction. Since the central nervous system of the upper limbs is closer to the brain and the movement of the upper limbs is more variable, most stroke patients are unable to control their limbs effectively, especially the upper limbs. Clinical practice has shown that the most effective way to treat hemiplegia is to stimulate the regeneration of the injured central nerves through repeated rehabilitation exercises [1,2].

The effectiveness of traditional rehabilitation therapy relies heavily on the expertise and knowledge of the rehabilitation practitioner [3]. Therefore, the use of robotics in the field of rehabilitation should take advantage of its strengths in performing high-intensity repetitive movements to alleviate muscle atrophy and joint stiffness [4,5,6]. The shortcomings of traditional rehabilitation training, such as a not obvious rehabilitation effect, high cost, and difficult operation, have been overcome by robot-assisted training [7]. In general, assisted training robots can be divided into two types: exoskeleton and end traction [8]. The representative upper limb rehabilitation robots CADEN-7 [9], EUROExos [10], and BONES [11,12] were mainly rope-driven or pneumatic muscle-driven exoskeleton-type robots, which can realize multi-joint rehabilitation training. Exoskeletal rehabilitation devices are complex and inconvenient to wear and are not suitable for small medical institutions such as clinics and homes. Zhang designed an end-guided upper limb rehabilitation robot, UECM [13], which can realize shoulder adduction/abduction and elbow flexion/extension movements in the horizontal plane. Yong had developed an end-guided upper limb rehabilitation robot that can simultaneously perform different modes of rehabilitation training for the right and left hands [14]. The end-effector robot transmits force through the grip and the user only needs to hold the grip to feel the assist or resistance, making it easy to get on and off the robot and change patients. Therefore, in the current situation of high demand for rehabilitation and patients queuing for treatment, the end-traction robot is an internationally recognized efficient design.

Currently, electric motors are mainly used as a rigid power source in rehabilitation robots. Due to the sudden change in load, the electric motor may stall, and the current will increase rapidly. In addition, there is a braking delay in the electric motor, which is a potential safety threat prone to causing secondary injuries to the patient. The use of smart materials in the robot transmission structure [15,16] can achieve better control during use [17,18]. Magnetorheological fluid (MRF) is a smart material consisting of soft magnetic material particles, carrier fluid, and surfactant, and its fluid properties can be changed reversibly according to the external magnetic field [19,20]. MRFs have the advantages of adjustable damping, a wide dynamic range, and a fast response time [21,22]. Because of their variable damping, ease of control, and low cost, magnetorheological devices are beginning to be used in rehabilitation equipment to provide flexible and reliable impedance to help patients recover [23,24,25].

Many scholars have researched and explored the use of MRF in the field of rehabilitation. Xu designed and studied a magnetorheological multimodal lower limb rehabilitation robot [26,27], which realized adaptive rehabilitation training based on human intention. In addition, magnetorheological devices were used in the lower limb ankle joints to assist the user in movements such as walking, jumping, and landing [28]. The MR Brake for wrist rehabilitation device developed by M. Avraam et al. [29] can achieve pronation/pronation movement of the wrist according to the set mode. The magnetorheological device designed by Takehito Kikuchi [30] and Noritaka Sato [31] for upper limb rehabilitation enables exercise recovery of upper limb muscles. The existing magnetorheological rehabilitation equipment can only provide rehabilitation control with fixed excitation current, but the motion of the hand is not fixed and random, which will cause fluctuations in the output damping torque. The magnetorheological joint damper developed in this paper can dynamically adjust the excitation current and robot speed according to the external motion state and the change in the control target and obtain a stable robot joint output torque.

The main purpose of this paper is to propose a magnetorheological upper limb rehabilitation training system. The structure of the paper is as follows: In Section 2, the mechanical mechanism of the robot trajectory and the MR joint damper are proposed, and the feasibility of the MR joint damper is verified by magnetic field simulation. In Section 3, the dynamic equation of the designed magnetorheological damper is established, and the accuracy of the dynamic equation is verified by experiments. In Section 4, the rehabilitation control strategy of the robot is introduced, the proposed control method is experimentally verified, and the safety performance of the rehabilitation robot is tested. In Section 5, the results are analyzed and discussed.

## 2. Robot Design

### 2.1. Mechanical Structure

**Figure 1:**
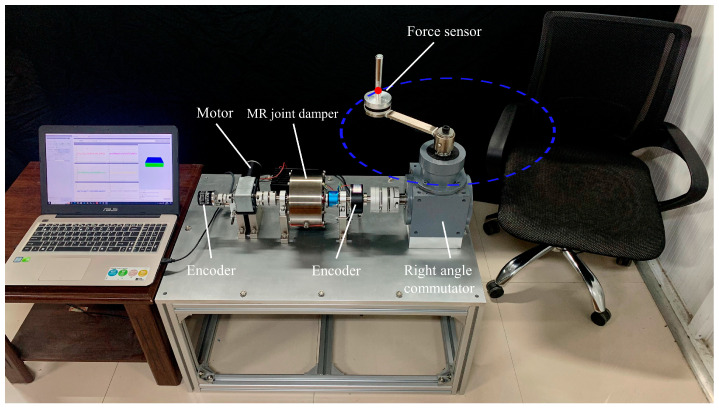
The prototype of the robot.

Circular movement of the upper limb in terminal traction is a multi-joint process that sequentially activates the muscles of the elbow and shoulder. In addition, 360° movement training of the arm in a circular motion requires coordination between the shoulder and elbow joints, which has a positive impact on upper limb rehabilitation training [31,32,33]. As shown in Figure 1, cyclic and repeated variable resistance exercises can positively improve upper limb mobility and help patients regain their athletic ability.

**Figure 2:**
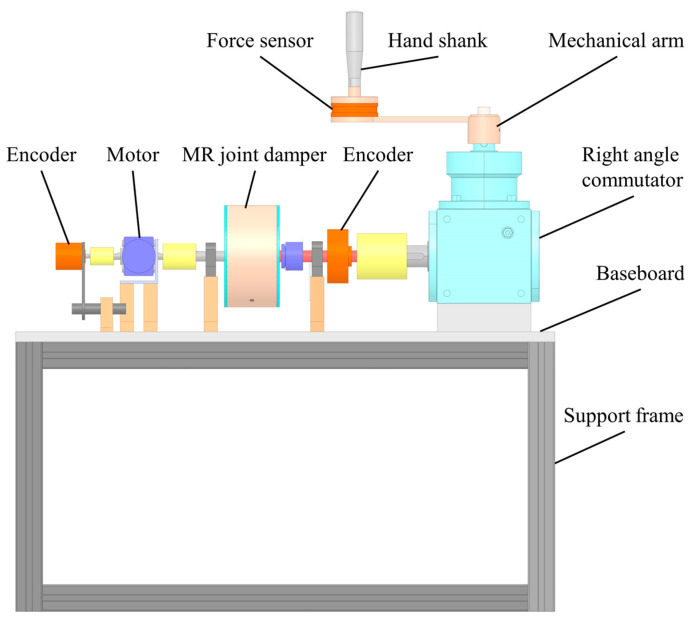
The design scheme of the robot.

**Figure 3:**
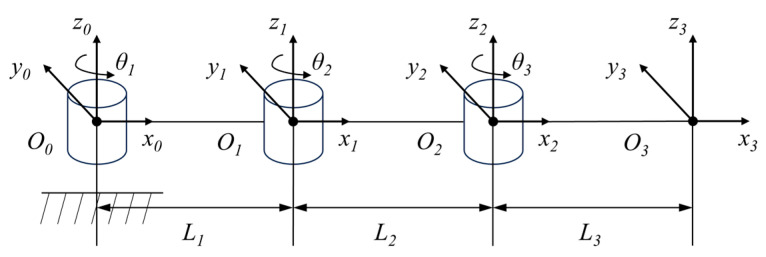
Coordinate diagram of each joint of the upper limb.

The mechanical structure of the upper limb rehabilitation device is shown in Figure 2. In the rehabilitation robot, it is powered by a decelerating motor and driven by an elastic coupling. The MR joint damper, as a power transmission mechanism, plays a role in adjusting the output interaction torque of rehabilitation robots in rehabilitation training. D–H parameter method is adopted to establish the horizontal coordinate system of the human upper limb with the shoulder joint as the origin, and the schematic diagram of the horizontal coordinate system of the upper limb is shown in Figure 3.

A kinematics analysis is to study the relationship between the end pose and joint angle. According to the homogeneous coordinate transformation equation, the pose matrix T30 of the end relative to the origin can be obtained (*cθ_i_* = *cosθ_i_*, *sθ_i_* = *sinθ_i_*):

(1) T30=[c(θ1+θ2+θ3)−s(θ1+θ2+θ3)0L1cθ1+L2c(θ1+θ2)+L3c(θ1+θ2+θ3)s(θ1+θ2+θ3)c(θ1+θ2+θ3)0L1sθ1+L2s(θ1+θ2)+L3s(θ1+θ2+θ3)00100001]

Based on the pose matrix, the equation for the coordinate position of the end in the plane is as follows:

(2) $$\{\begin{array}{c} x=L_{1}c\theta_{1}+L_{2}c\left(\theta_{1}+\theta_{2}\right)+L_{3}c\left(\theta_{1}+\theta_{2}+\theta_{3}\right)\\ y=L_{1}s\theta_{1}+L_{2}s\left(\theta_{1}+\theta_{2}\right)+L_{3}s\left(\theta_{1}+\theta_{2}+\theta_{3}\right)\\ z=0 \end{array}$$

Upper limb spatial position:

(3) $$R=\left[\begin{array}{ccc} c\left(\theta_{1}+\theta_{2}+\theta_{3}\right) & -s\left(\theta_{1}+\theta_{2}+\theta_{3}\right) & 0\\ s\left(\theta_{1}+\theta_{2}+\theta_{3}\right) & c\left(\theta_{1}+\theta_{2}+\theta_{3}\right) & 0\\ 0 & 0 & 1 \end{array}\right]$$

**Figure 4:**
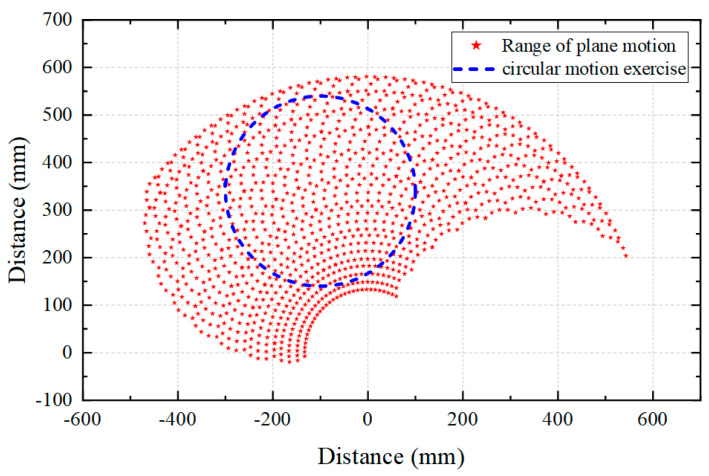
Range of upper limb motion trajectories.

Upper limb kinematics analysis has the potential to be a useful tool in clinical decision-making. The upper limb offers many degrees of freedom, coordinated movement across multiple joints, and a wide range of motion at the joints. As shown in Table 1, the range of motion and human body size required for daily activities by healthy adults are quantified. Based on this standard, we designed the trajectory and range of motion of the rehabilitation robot. By comparing the motion range of the human upper limb with the motion trajectory of the rehabilitation robot in Figure 4, the planned rehabilitation motion trajectory (radius R = 200 mm) conforms to the motion range of the upper limb, which can ensure the safety and rationality of rehabilitation training.

### 2.2. Magnetorheological Joint Damper Design and Simulation

Muscle strength is the force produced by muscles when they contract and are excited and is necessary for the body to maintain posture, initiate, or control movement. Shor-term and long-term treatment plans are developed based on muscle strength assessment results. The Lovett muscle strength rating scale is used to evaluate the effectiveness of functional recovery after stroke. The muscle strength rating scale is shown in Table 2. The maximum gripping force that can be generated by the upper limb is 400 N for healthy adult males and 228 N for females [35]. Considering that it is mainly intended for elderly patients, the rehabilitation device provides a maximum of 20 N of human–machine interaction force (about 10% of the force of a healthy handgrip). The end of the subject’s upper limb was set to perform a circular motion with a radius of R = 200 mm together with the rehabilitation device.

**Figure 5:**
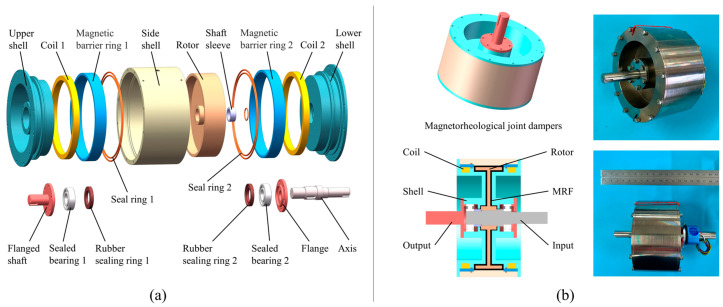
(**a**) Exploded view of MR joint damper; (**b**) MR joint damper model and real picture.

In a rehabilitation robot system, MR joint dampers generate controlled torque for the robot joints by adjusting the input current. As shown in Figure 5, the MR joint damper is mainly composed of magnetorheological fluid, rotor, stator, excitation coil, and sealing device. The specific design parameters of MR joint dampers are shown in Table 3. The magnetorheological fluid is evenly distributed in the gap between the rotor and the stator, and a magnetic field is generated around the exciting coil when a current is passed into the coil. Magnetorheological fluid changes from fluid form to a solid-like form under the action of a magnetic field. The T-type rotor designed in this paper combines the two ends of the rotor and the cylinder of the rotor as the effective working area, which has the advantages of a small volume, compact structure, and large damping torque.

**Figure 6:**
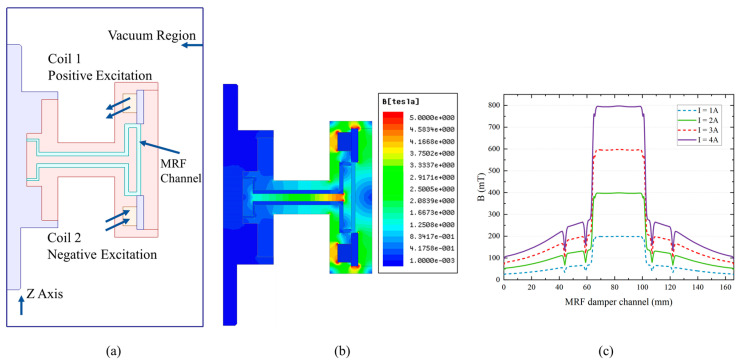
Electromagnetic simulation of MR joint damper. (**a**) Simulation setting. (**b**) Cloud image of magnetic field distribution. (**c**) The magnetic field strength of the damping channel.

The electromagnetic simulation of MR joint dampers is carried out to verify the rationality of the structural design. Figure 6 shows the simulation of the magnetic field when the maximum excitation current (2 A) is applied. In the magnetic field cloud image, we can see that the magnetic field becomes stronger the closer you get to the coil. The magnetic field generated by the upper and lower coils is symmetrically distributed, and the two magnetic fields are superimposed on each other at the rotor to obtain a greater magnetic field effect. We can observe in more detail the magnetic field changes in the damped channels filled with magnetorheological fluids under different excitation currents (1 A, 2 A, 3 A, and 4 A). The gap width of the magnetorheological liquid filled in the shell is δ, $R_{1}$ and $R_{2}$ are the inner and outer diameters of the effective ring on the end face, H is the magnetic field intensity generated by the excitation coil, h is the height of the ring, $\tau_{m}$ is the static yield stress, and η is the apparent viscosity of the magnetorheological liquid. According to the Bingham model, the output torque of MR joint dampers can be calculated as follows:

(4) $$M=M_{d}+M_{Z}$$

$M_{d}$ is the torque transmitted by the end face of the rotor, and $M_{z}$ is the torque transmitted by the circumferential surface of the rotor:

(5) $$M_{d}=\overset{R_{2}}{\underset{R_{1}}{\int }}2\pi r^{2}\left[\tau_{m}\left(H\right)+\eta \frac{r\left(\omega_{1}+\omega_{2}\right)}{\delta }\right]dr=\frac{2}{3}\pi \left(R_{2}^{3}-R_{1}^{3}\right)\tau_{m}\left(H\right)+\frac{\pi }{2\delta }\eta \left(\omega_{1}+\omega_{2}\right)\left(R_{2}^{4}-R_{1}^{4}\right)$$

(6) $$M_{Z}=\tau AR=2\pi r^{2}h\left[\tau_{m}\left(H\right)+\eta \frac{r\left(\omega_{1}+\omega_{2}\right)}{\delta }\right]$$

It can be seen from the magnetic field simulation results that the magnetic field distribution in the working area of the damper is reasonable, and the magnetorheological field in the damping channel can realize the role of transmitting damping torque. The relationship between the excitation current and magnetic induction intensity can be obtained using a simulation. Under normal circumstances, when the working current of the MR joint damper is 0–2 A, the output torque is 0–5 NM, so we can provide 0–25 N pressure for the rehabilitation training of the limbs.

### 2.3. MR Joint Damper Dynamic Model

To obtain a good control effect, the establishment of an accurate dynamic model is a prerequisite for achieving good results. MR joint dampers have strong nonlinear and hysteretic properties, which make their accurate modeling complicated. The hyperbolic tangent model [37] can describe the hysteretic characteristics of MRF, which can be expressed as follows:

(7) $$\{\begin{array}{c} F=c\dot{\theta }+k\theta +\alpha z+f_{0}\\ z=\tanh\left(\beta \dot{\theta }+\delta sign\left(\theta \right)\right) \end{array}$$

where *c* is the damping coefficient, *k* is the stiffness coefficient, *α* denotes the proportionality coefficient related to the hysteresis characteristic, *β* denotes the proportionality coefficient related to the slope of the hysteresis curve, *δ* denotes the half-width of the hysteresis curve, and $f_{0}$ is the bias force.

**Figure 7:**
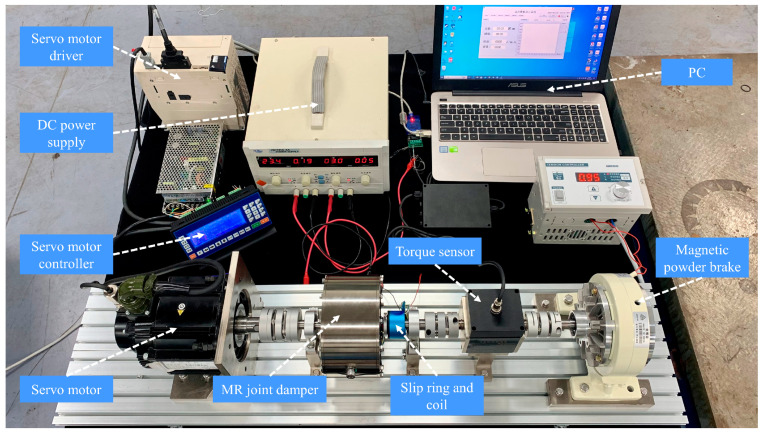
The experimental device tests the relationship between the torque of MR joint damper and the current and speed.

The calibration test platform of the MR joint damper is shown in Figure 7. The servo motor (Yaskawa Motor SGM7G20AFC61) generates the driving force, and the dynamic torque sensor (Zhongwan Jinno JN-DN5) is installed on the output shaft of the MR joint damper. The adjustable current input to the coil is provided by a DC power supply. The end payload is regulated by the magnetic powder brake (Jiangsu Haowen PB-12). During the test, the current (0–4 A) and the motor (amplitude ±10° frequency 1 Hz) are adjusted to achieve different output torques of the MR joint dampers.

The experimental data on the relationship between the damping torque of different currents and displacement and velocity are used as the model training data. The parameters of the hyperbolic tangent model are identified by genetic algorithm and least square method [38]. The set parameters *c*, *k*, *α*, *β*, and *δ* are current-dependent parameters, and a polynomial in the current is fitted to the parameters at different currents as follows:

(8) $$\{\begin{array}{c} c=a_{1}I^{3}+a_{2}I^{2}+a_{3}I+a_{4}\\ k=a_{5}I^{4}+a_{6}I^{3}+a_{7}I^{2}+a_{8}I+a_{9}\\ \alpha =a_{10}I^{4}+a_{11}I^{3}+a_{12}I^{2}+a_{13}I+a_{14}\\ \beta =a_{15}I^{4}+a_{16}I^{3}+a_{17}I^{2}+a_{18}I+a_{19}\\ \delta =a_{20}I^{4}+a_{21}I^{3}+a_{22}I^{2}+a_{23}I+a_{24} \end{array}$$

The results of parameter identification are obtained as shown in Table 4.

**Figure 8:**
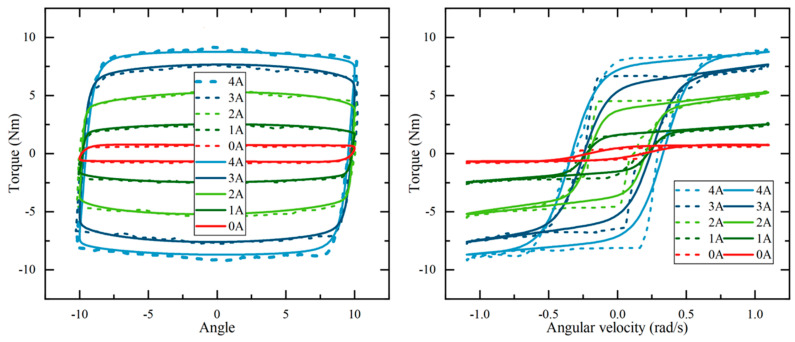
Comparison between model and experiment (dashed line is experimental data; solid line is function fitting curve).

Comparing the established hyperbolic tangent model with the experimental data when the excitation signal has an amplitude of ±10° at a frequency of 1 Hz, and a comparison of the model fit is shown in Figure 8. It can be found that the established hyperbolic tangent model fits the experimental results well. This reflects the velocity characteristics and hysteresis characteristics of the damping torque of the MR joint damper. At the same time, the validity of the model is verified. The damping force increases with the increase in current. To obtain a better control effect, we mainly chose a 0–2 A excitation current for the experiment.

The damping torque output of the MR joint damper is related to the rotation angle and angular velocity of the damper connecting rod and the input control current. In the actual engineering process, it is often necessary to deduce the corresponding control current according to the expected damping torque and the current motion state. Due to the nonlinear characteristics of the MR devices, the inverse mechanical model of the MR joint damper can be trained by a BP neural network through the obtained hyperbolic tangent model. The input layer and output layer of the BP neural network are set as follows:

(9) $$x=\left[\begin{array}{c} x_{1}=velocity\\ x_{2}=Desired\;damping\;moment \end{array}\right]$$

(10) $$y=\left[y_{1}=excitation\;current\right]$$

## 3. Robot System Implementation

**Figure 9:**
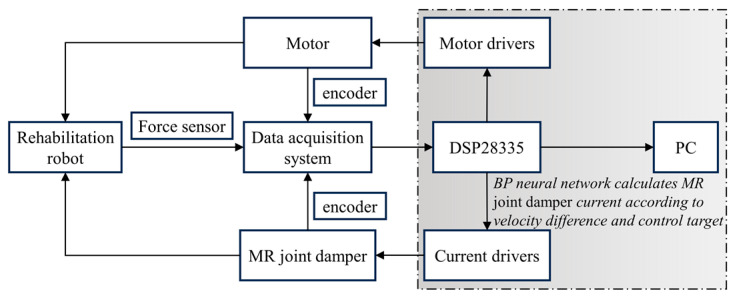
Hardware composition of the control system.

Figure 9 shows the hardware composition of the rehabilitation robot control system. The DSP28335 chip is the host computer’s main control chip to undertake the main data processing and program control functions. The encoder is selected as an extended-range encoder with a resolution of 1024 pulse signals per revolution. The torque sensor is located below the handle and is used to monitor the interaction torque between the feedback patient and the rehabilitation robot. The motion data acquisition system is responsible for collecting the motion speed of human upper limbs, motor speed, and human–computer interaction torque and transmitting them to the main control chip. The motor and current driver execute the program command to dynamically adjust the motor speed with the current of the MR joint damper through the PWM signal.

**Figure 10:**
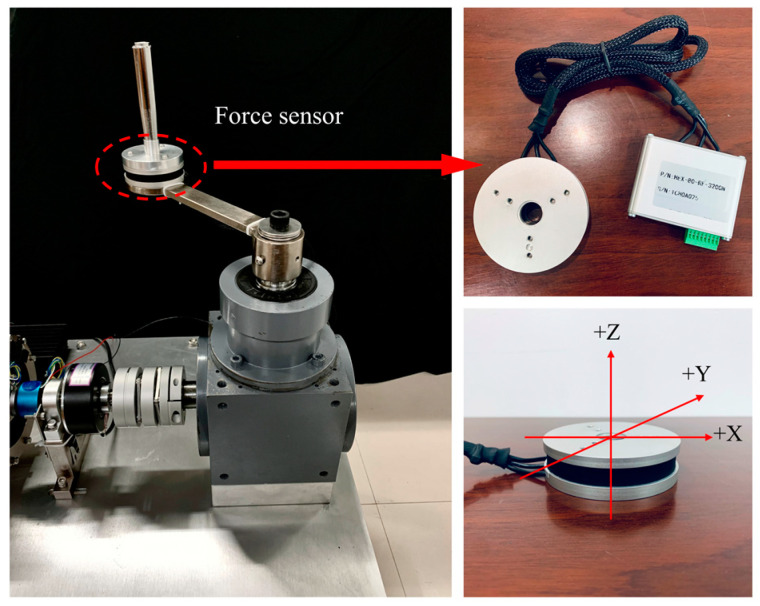
Interactive torque data acquisition.

As shown in Figure 10, the robotic arm transmits the torque of the MR joint damper to the affected limb through the handle. The three-dimensional force sensor (HEX80RE3200N) will be responsible for real-time monitoring of the human–computer interaction torque.

## 4. Experiment Results and Discussion

### 4.1. Training Controller

In the treatment and rehabilitation of traditional hemiplegia patients, exercise training of the affected limb plays an important role. Rehabilitation training requires many repetitive movements to rebuild the motor nerves while ensuring that the auxiliary force provided remains stable. The training controller of the rehabilitation robot is designed for this goal. MR joint dampers accurately provide auxiliary force to the patient when the strength is insufficient, ensuring the completion of the training goal, and are suitable for establishing the motor coordination ability of the affected limb.

Establish a mechanical model of human–computer interaction:

(11) $$M\ddot{x}+B\dot{x}+Kx=\tau_{rob}+\tau_{MR}$$

where $\ddot{x,}\dot{x,}x$ represent the acceleration, velocity, and displacement of the human–robot interaction end; *M*, *B*, and *K* denote the inertia, damping, and stiffness of the system, respectively; $\tau_{rob}$ denotes the human–robot interaction torque of the robotic system; and $\tau_{MR}$ denotes the torque supplied by the MR joint damper.

**Figure 11:**
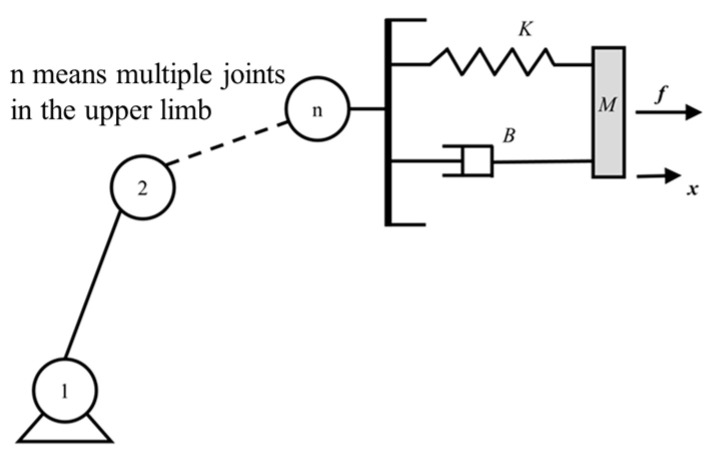
Mass block-spring-damping system.

As shown in Figure 11, the interaction between the robot and the environment in the supple state can be viewed as a mass block-spring-damping second-order system. The robot joint torque is adjusted according to the deviation of the actual position from the set position to achieve tracking control of torque and velocity.

**Figure 12:**
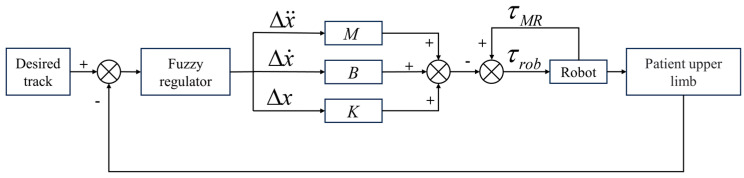
Control strategy block diagram.

As shown in Figure 12, a block diagram of the training control strategy is presented. In the process of training control, the upper limb has a poor movement ability to drive the rehabilitation robot. When the sensor detects slight movement, the motor drives the MR joint damper at a faster speed to provide positive rehabilitation force in the same direction. Currently, the stable output of the end interaction force of the rehabilitation robot is realized by dynamically adjusting the current of the MR joint damper.

### 4.2. Training Performance Testing

The main purpose of the upper limb rehabilitation equipment controller is to realize the variable damping rehabilitation training device by adjusting the excitation current of the MR joint damper. The dynamic model of the MR joint damper is established by experiments, and the reverse dynamic model of the MR joint damper is obtained using the BP neural network calculation method and the forward dynamic model. The end of the subject’s upper limb is set to perform a circular motion with a radius of R = 200 mm together with the rehabilitation equipment, and the subject’s upper limb mass is about 5 kg.

**Figure 13:**
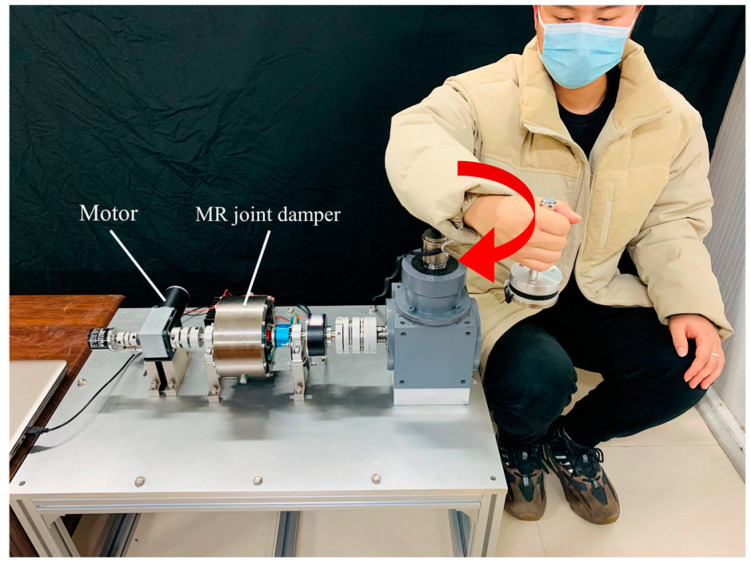
Motion diagram.

In the experiment, the affected limb of the tester was relaxed on the handle. When the robot detected a slight movement speed of the hand, the handle of the rehabilitation device began to drive the affected limb to move, as shown in Figure 13.

**Figure 14:**
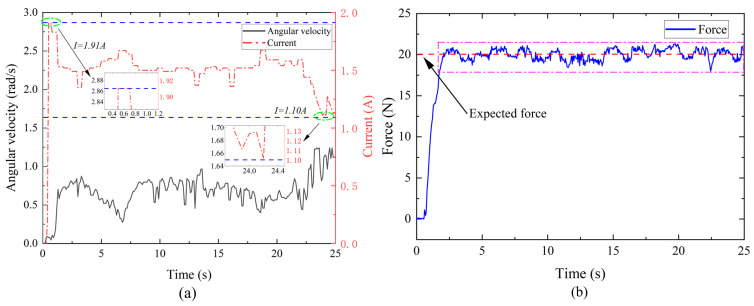
Upper limb rehabilitation exercise. (**a**) Velocity change and current change; (**b**) the end sensor monitors the change in the human–computer interaction force.

Since the velocity of the rehabilitation motion of the hand is random and uncertain, the velocity difference between the input end and the output end of the MR joint damper changes in real time. By monitoring the change in velocity differential, the excitation current is adjusted in real time according to the reverse dynamic model of the MR joint damper and the human–computer interaction model, to realize stable tracking of the end force output. As shown in Figure 14a, the speed of the affected limb varies randomly during rehabilitation exercise, during which the excitation current varies in real time from a maximum of 1.91 A to a minimum of 1.10 A (in line with the given current range 0–2 A). As shown in Figure 14b, the end sensor monitors the change in the human–computer interaction force during the operation of the rehabilitation device. The expected torque is set to 20 N, the average output expected torque is 20.02 N, and the standard deviation is 0.635 N during operation. The output torque has good stability.

**Figure 15:**
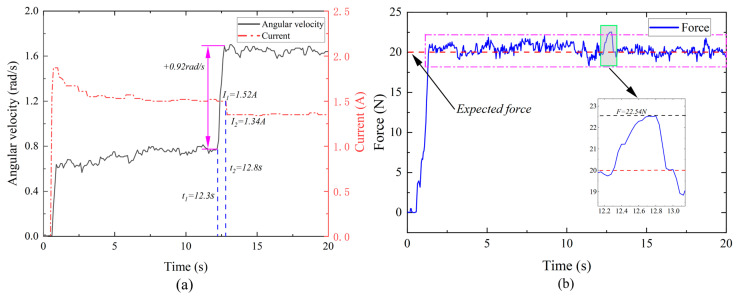
Safety testing. (**a**) Velocity change and current change; (**b**) the end sensor monitors the change in the human–computer interaction force.

Compared with common motor control, there are hidden safety problems caused by the stalling impact. The upper limb recovery device used for rehabilitation pays more attention to safety considerations. The safety test results of the upper limb rehabilitation robot in the event of a sudden motor stall (speed mutation increase) are shown in Figure 15. As shown in Figure 15a, the motor speed changes during normal operation, and the controller detects the change in speed within a short period of time (0.5 s) and adjusts the excitation current (from 1.52 A to 1.34 A). When the motor speed changes abruptly, the end output torque increases by 12.5% and quickly recovers to the expected torque, as shown in Figure 15b. In the process of safety testing, the average value of the output expected torque is 20.38 N and the standard deviation is 0.645 N, which can maintain the stability of the output torque.

## 5. Conclusions

To help stroke patients with upper limb movement disorders recover their movement ability, a rehabilitation training device and control method based on magnetorheological joint dampers are designed in this paper. To meet the control requirements, a dynamic model of the magnetorheological joint damper is established, and an experimental platform for the dynamic model is set up. The hyperbolic tangent model of the magnetorheological joint damper is obtained by a genetic algorithm and the least square method, and the inverse dynamic model is obtained by a BP neural network. The establishment of a dynamic model provides a basis for further research on the performance and control of rehabilitation devices. The joint damper based on MR fluid can realize the characteristics of adjustable and controllable damping torque and output damping torque 0–5.0 Nm under the condition of working current (0–2 A), which can meet the needs of dynamic adjustment of human–computer interaction force when recovering patients perform recovery exercise. In this paper, the control strategy of the rehabilitation robot is tested experimentally, and the specific results are as follows:

(1) The rehabilitation-compliant joint based on MR fluid can dynamically adjust the excitation current according to the changes in the motion state of the human upper limb. The expected torque is set to 20 N, and the average value of the output expected torque is 20.02 N, and the standard deviation is 0.635 N during the random motion of the upper limb velocity. The output torque has good stability.
(2) The application of MR devices in rehabilitation training improves flexibility and safety compared with traditional motor drives. It overcomes the shortcomings of the traditional rehabilitation device, which is easy to have impact and unstable human–computer interaction force causing secondary injury. The motor speed changes during normal operation, and the controller detects the change in speed for a short period of time (0.5 s) and adjusts the excitation current (from 1.52 A to 1.34 A). When the motor speed changes, the end output torque increases by 12.5% and quickly recovers to the desired torque. In the process of safety test, the average value of the output expected torque is 20.38 N and the standard deviation is 0.645 N, which can still maintain the stability of the output torque.

Because the speed of upper limb rehabilitation movement is a random speed and there is a delay problem in the control of MR devices, there is a problem of fluctuations in the end human–computer interaction torque. To achieve better control results, further research will be carried out, for example, by providing linear motors to help patients move smoothly.

## Figures and Tables

**Table 1:** Parameter of upper limb motion [34].

Joint	Degrees of Freedom	Range	Length (mm)
Shoulder joint	horizontal abduction–adduction	−65°~105° (*θ*_1_)	330 (*L*_1_)
Elbow joint	flexion–extension	0°~141° (*θ*_2_)	255 (*L*_2_)
Wrist joint	flexion–extension	−40°~38° (*θ*_3_)	50 (*L*_3_)

**Table 2:** The muscle strength rating scale [36].

Rank	Name	Standard	Compared to Normal
0	None	No muscle contraction	0%
1	Weak	There is slight contraction, but no joint movement	10%
2	Poor	Ability to do full range of motion of joints in a reduced weight state	25%
3	Fair	Full range of joint motion against gravity, but not resistance	50%
4	Fine	Resist gravity and certain resistance, and do full range of joint movement	75%
5	Normal	Resist gravity and sufficient resistance, do full range of joint movement	100%

**Table 3:** MR joint damper specifications.

Parameter	Value	Material
MR joint damper diameter	151 mm	/
MR joint damper width	82 mm	/
MR joint damper mass	6.9 kg	/
Channel gap	2 mm	/
Flanged shaft length	90 mm	304 stainless steels
Axis length	122.70 mm	304 stainless steels
Upper shell width	37 mm	Q235
Lower shell width	37 mm	
Side shell diameter	151 mm	Q235
Side shell width	74 mm	
Rotor diameter	130 mm	Q235
Rotor width	34 mm	
Magnetic barrier ring diameter	130 × 137 mm	6061 aluminum alloy
Magnetic barrier ring width	18 mm	
Wire diameter	0.59 mm	Copper
Coil turns	100 × 2	

**Table 4:** MR joint damper hyperbolic tangent model parameter identification results.

Parameter	Value	Parameter	Value	Parameter	Value
$a_{1}$	0.04536	$a_{10}$	−2.486	$a_{19}$	3.686
$a_{2}$	−0.02071	$a_{11}$	18.6	$a_{20}$	−1.214
$a_{3}$	−0.8984	$a_{12}$	−42.14	$a_{21}$	9.759
$a_{4}$	0.03166	$a_{13}$	28.34	$a_{22}$	−24.39
$a_{5}$	0.002454	$a_{14}$	−0.7454	$a_{23}$	18.87
$a_{6}$	−0.01988	$a_{15}$	5.258	$a_{24}$	−0.7968
$a_{7}$	0.0447	$a_{16}$	−42.56	$f_{0}$	0.03472
$a_{8}$	−0.01778	$a_{17}$	106.9		
$a_{9}$	−0.0058	$a_{18}$	−82.78		

## Data Availability

The original contributions presented in the study are included in the article, and further inquiries can be directed to the corresponding author.

## References

[1] Germanotta M., Cruciani A., Galli C., Cattaneo D., Spedicato A., Aprile I. (2020). Time course of the upper limb motor recovery in subacute stroke patients undergoing conventional or robotic rehabilitation. J. Biol. Regul. Homeost. Agents.

[2] Irisawa H., Mizushima T. (2020). Correlation of Body Composition and Nutritional Status with Functional Recovery in Stroke Rehabilitation Patients. Nutrients.

[3] Huo W., Mohammed S., Moreno J.C., Amirat Y. (2016). Lower Limb Wearable Robots for Assistance and Rehabilitation: A State of the Art. IEEE Syst. J..

[4] Maciejasz P., Eschweiler J., Gerlach-Hahn K., Jansen-Troy A., Leonhardt S. (2014). A survey on robotic devices for upper limb rehabilitation. J. Neuroeng. Rehabil..

[5] Leonardis D., Barsotti M., Loconsole C., Solazzi M., Troncossi M., Mazzotti C., Castelli V.P., Procopio C., Lamola G., Chisari C. (2015). An EMG-Controlled Robotic Hand Exoskeleton for Bilateral Rehabilitation. IEEE Trans. Haptics.

[6] Uchida M., Morita Y., Magasaki M., Ukai H., Matsui N. (2011). Development of rehabilitation training support system of upper limb motor function for personalized rehabilitation. Int. J. Appl. Electromagn. Mech..

[7] Sheng B., Zhang Y.X., Meng W., Deng C., Xie S. (2016). Bilateral robots for upper-limb stroke rehabilitation: State of the art and future prospects. Med. Eng. Phys..

[8] Yang Q., Cao D., Zhao J. (2013). Analysis on State of the Art of Upper Limb Rehabilitation Robots. Robot.

[9] Perry J.C., Rosen J., Bums S. (2007). Upper-limb powered exoskeleton design. IEEE Asme Trans. Mechatron..

[10] Vitiello N., Lenzi T., Roccella S., De Rossi S.M., Cattin E., Giovacchini F., Vecchi F., Carrozza M.C. (2013). NEUROExos: A Powered Elbow Exoskeleton for Physical Rehabilitation. IEEE Trans. Robot..

[11] Klein J., Spencer S.J., Allington J., Minakata K., Wolbrecht E.T., Smith R., Bobrow J.E., Reinkensmeyer A.D. Biomimetic Orthosis for the Neurorehabilitation of the Elbow and Shoulder (BONES). Proceedings of the 2008 2nd IEEE RAS & EMBS International Conference on Biomedical Robotics and Biomechatronics.

[12] Klein J., Spencer S., Allington J., Bobrow J.E., Reinkensmeyer D.J. (2010). Optimization of a Parallel Shoulder Mechanism to Achieve a High-Force, Low-Mass, Robotic-Arm Exoskeleton. IEEE Trans. Robot..

[13] Zhang Y.B., Wang Z.X., Ji L.H., Bi S. The clinical application of the upper extremity compound movements rehabilitation training robot. Proceedings of the 2005 IEEE 9th International Conference on Rehabilitation Robotics.

[14] Yang Y., Wang L., Tong H., Zhang L. Arm rehabilitation robot impedance control and experimentation. Proceedings of the 2006 IEEE International Conference on Robotics and Biomimetics.

[15] Christie M.D., Sun S., Deng L., Du H., Zhang S., Li W. (2023). Shock Absorption for Legged Locomotion through Magnetorheological Leg-Stiffness Control. Machines.

[16] Lu Y., Cao Y., Chen Y., Li H., Li W., Du H., Zhang S., Sun S. (2023). Investigation of a wearable piezoelectric-IMU multi-modal sensing system for real-time muscle force estimation. Smart Mater. Struct..

[17] Ma H., Chen B., Qin L., Liao W.H. (2017). Design and testing of a regenerative magnetorheological actuator for assistive knee braces. Smart Mater. Struct..

[18] Huang L.J., Hu H.S., Ouyang Q. (2022). Design and Feasibility Study of MRG-Based Variable Stiffness Soft Robot. Micromachines.

[19] Liu G.S., Hu H.S., Ouyang Q., Zhang F. (2023). Multi-Objective Optimization Design and Performance Comparison of Magnetorheological Torsional Vibration Absorbers of Different Configurations. Materials.

[20] Ouyang Q., Hu H.S., Qian C., Zhang G., Wang J., Zheng J. (2019). Investigation of the Influence of Magnetic Field Distribution on the Magnetorheological Absorber With Individually Controllable Coils. IEEE Trans. Magn..

[21] Andrade R.M., Bento A., Vimieiro C.B.S., Pinotti E.M. (2018). Optimal design and torque control of an active magnetorheological prosthetic knee. Smart Mater. Struct..

[22] Deng L., Sun S.S., Christie M., Ning D., Jin S., Du H., Zhang S., Li W. (2022). Investigation of a seat suspension installed with compact variable stiffness and damping rotary magnetorheological dampers. Mech. Syst. Signal Process..

[23] de Andrade R.M., Ulhoa P.H.F., Dias E.A.F., Filho A.B., Vimieiro C.B. (2023). Design and testing a highly backdrivable and kinematic compatible magnetorheological knee exoskeleton. J. Intell. Mater. Syst. Struct..

[24] Liu G.Y., Gao F., Wang D.H., Liao W.H. (2022). Medical applications of magnetorheological fluid: A systematic review. Smart Mater. Struct..

[25] Wang D.M., Wang Y.K., Zi B., Cao Z., Ding H. (2020). Development of an active and passive finger rehabilitation robot using pneumatic muscle and magnetorheological damper. Mech. Mach. Theory.

[26] Xu J.J., Li Y.F., Xu L.S., Peng C., Chen S., Liu J., Xu C., Cheng G., Xu H., Liu Y. (2019). A Multi-Mode Rehabilitation Robot with Magnetorheological Actuators Based on Human Motion Intention Estimation. IEEE Trans. Neural Syst. Rehabil. Eng..

[27] Cheng G.X., Xu L.S., Xu J.J., Liu J., Shi J., Chen S., Liu L., Liang X., Liu Y. (2021). Robotic mirror therapy system for lower limb rehabilitation. Ind. Robot. Int. J. Robot. Res. Appl..

[28] Khazoom C., Veronneau C., Bigue J.P.L., Grenier J., Girard A., Plante J.S. (2019). Design and Control of a Multifunctional Ankle Exoskeleton Powered by Magnetorheological Actuators to Assist Walking, Jumping, and Landing. IEEE Robot. Autom. Lett..

[29] Avraam M., Horodinca M., Romanescu I., Preumont A. (2010). Computer Controlled Rotational MR-brake for Wrist Rehabilitation Device. J. Intell. Mater. Syst. Struct..

[30] Kikuchi T., Otsuki K., Furusho J., Abe H., Noma J., Naito M., Lauzier N. (2010). Development of a Compact Magnetorheological Fluid Clutch for Human-Friendly Actuator. Adv. Robot..

[31] Abdelhameed E.H., Sato N., Morita Y. Design of Variable Resistance Training System Using Rotary Magnetorheological Brake Ataxic Patients’ Upper Limb Rehabilitation. Proceedings of the 3rd IEEE International Conference on Control, Automation and Robotics.

[32] Bosga J., Meulenbroek R.G.J., Swinnen S.P. (2003). Stability of inter-joint coordination during circle drawing: Effects of shoulder-joint articular properties. Hum. Mov. Sci..

[33] Zhang S., Zuo G., Shi C., Xu J., Liu X., Gao J., Li G. The sEMG characteristics of human upper limb during circle drawing on EULRR system. Proceedings of the IEEE International Conference on Cybernetics and Intelligent Systems (CIS) IEEE Conference on Robotics, Automation and Mechatronics (RAM).

[34] Gates D.H., Walters L.S., Cowley J., Wilken J.M., Resnik L. (2016). Range of Motion Requirements for Upper-Limb Activities of Daily Living. Am. J. Occup. Ther..

[35] Khanicheh A., Muto A., Triantafyllou C., Weinberg B., Astrakas L., Tzika A., Mavroidis C. (2006). fMRI-compatible rehabilitation hand device. J. Neuroeng. Rehabil..

[36] Ren Z., Ye S., Nie Q., Feng J., Liu K., Li Q., Wen J. (2023). Application of digitization and visualization-based muscle strength measurement in ischemic stroke patients with motor dysfunction. Sci. Rep..

[37] He W., Ouyang Q., Hu H.S., Ye X., Lin L. (2022). Semi-active control of crankshaft skyhook based on magnetorheological torsional damper. Front. Mater..

[38] Zhu H., Rui X., Yang F., Zhu W., Wei M. (2019). An efficient parameters identification method of normalized Bouc-Wen model for MR damper. J. Sound Vib..

